# The expanding CML treatment landscape: an introspective commentary

**DOI:** 10.1038/s41408-023-00918-3

**Published:** 2023-09-13

**Authors:** Jeffrey H. Lipton

**Affiliations:** Leukemia Group, Princess Margaret Cancer Centre, University of Toronto, Toronto, Canada

**Keywords:** Medical research, Leukaemia

The article by Abdelmagid and co-authors on real-world experience with ponatinib that accompanies this editorial is interesting. There are three points to take from this. First, it confirms the number of articles coming out, not just ponatinib but also all TKIs, that suggest dose reduction will reduce adverse events without losing response. The second of course, is that results with real-world therapy are often not quite as good as those with closely- monitored controlled studies, but are often very reasonable. The third is that previous therapy may very well impact the current therapy, both in terms of efficacy and side effects and needs to be taken into consideration when choosing the best drug.

Chronic myeloid leukemia is a disease which has changed dramatically at multiple levels over the last 50 years. There have been many reviews written about developments in understanding the biology, the disease monitoring and therapy that have propelled this disease to be the poster child for other malignant diseases [1]. Other than her2 in breast cancer, this is the first example of rationally developed targeted therapy. I do not want to go here in this review, where others have gone. perhaps it should instead be called an introspective commentary. If that is what the reader is looking for, then let us go on. I will deal with chronic phase disease only. This commentary is by no means exhaustive and I will cite papers to give a flavor to the points raised. I mean no disrespect to the authors of those not included. And for those that know me, this is about as woke as I get.

As background, I am an old timer in the CML field. I started out as a stem cell transplanter who would also would tolerate patient complaints about fatigue and depression on interferon, tiptoed with uncertainty into the imatinib era and eventually calling it home. As a baseline, when we started our first STI571 (imatinib) trial, I saw around 120 new cml patients in the first three months who were sitting around on palliative hydroxyurea ineligible for transplant because of age (< 40), absence of matched related donors, co-morbidities or unable to manage the side effects of interferon.

In the olden days, aka pre imatinib, the only curative treatment for CML was allogeneic bone marrow transplant (BMT) [2] and in some cases alpha-interferon (IFN) [3]. In the case of BMT, there were age limitations of around 40, having a full sibling match and being healthy enough to stand the procedure. Survival rates in good centers approached 90% but not without the possibility of long-term irreversible side effects. Times have changed. We can transplant patients to ages into the seventies, with matched, mismatched related donors, unrelated matched or mismatched donors, cord blood, and in recent years haplo-identical donors. HLA-typing is more precise, graft-versus-host-disease prophylaxis and therapy are better and supportive care is better to the point where patients with less robust performance may still be candidates. IFN therapy was much less effective and caused many issues in patients especially as they became older. With the transition to standard tyrosine kinase therapy, transplant almost disappeared as a treatment and now even if appropriate for TKI intolerant or resistant patients, is often forgotten until the disease has progressed and results are not as good.

With TKIs, starting with first generation imatinib, second generation dasatinib, nilotinib, radotinib, and bosutinib, third generation ponatinib and alternate site targeted drugs such as asciminib virtually everybody can be treated successfully to the point that survival of compliant patients is essentially that of age-matched controls. In some cases patients can be cured, i.e. come off therapy, or achieve in the words of the late John Goldman, a functional cure, meaning they can live on therapy until they die from something else. Oh yes, some patients are shocked to find out that they did not achieve immortality [4].

What has been shown however, is that moving to newer and “more powerful” drugs in first line has not improved the survival beyond that which was achieved with first generation imatinib [5]. Perhaps a few more patient will achieve the starting point for considering a try for TFR, but the current success rate for TFR in most studies, still hovers around 50% regardless of which drug is used [6, 7]. There is optimism that with longer therapy, more patients will achieve successful TFR in the future. Predicting prognosis at diagnosis more specifically may help select the potentially successful candidates [8].

It is also interesting that survival generally is almost the same if a patient achieves a stable 2-log (1% IS or complete cytogenetic remission equivalence) when compared to a deep molecular remission (DMR), defined as a 4.5-log or greater (0.0032% IS) [5].

There has been some down sides to moving beyond imatinib. Although imatinib for the most part was associated with chronic, nagging side effects, the next generations have been associated with more severe side effects, particularly in the area of arterial vascular events, not always predictable when starting a newer drug or switching to a newer drug from imatinib when trying to deepen the response [9]. Despite this, newer drugs can be extremely effective in overcoming resistant disease, but in the context of balancing efficacy against adverse events [10–12]. There is much interest in the dose reduction strategy for induction or even maintenance for people who will be on long-term therapy [13, 14].

The other major “side effect” is not quantified in any common toxicity grading scale. I am referring to the elephant in the room, the economic or financial adverse event. An excellent ASCO web-cast by a fervent cost control champion, Hagop Kantarjian [15], shows that drug costs have exploded from a hundred dollars a month or even less for generic imatinib to tens of thousands of dollars a month for the newest drugs. Globally, this means that many of these drugs will not be available outside first world countries. It also means that the financial burden on patients with normal life spans, third party payers or socialized society payers, gets heavier and heavier and is cumulative. And what has been gained for this accelerating cost cycle? A few more TFR eligible patients. No increase in survival, no increase in the percentage of successful TFR as yet. Just an increase in more serious toxicity. Also as we use up the more powerful drugs earlier on, either by starting on them or switching early or frequently, we will lose our safety net for later down the road for the minority of patients who truly are intolerant or resistant. And remember, allografting is not even on the radar for most physicians until late when disease may have progressed, and as a result is less successful.

So what am I saying? Heaven forbid not to discontinue research and development in CML. The new drugs have helped rescue the early failures in many cases and are most welcome. Do we need them first line? On rare occasions yes – for higher risk patients, for those who want a better chance of getting off therapy such as young pregnancy-aged women for example. New strategies to find ways of getting at the CML stem cell and eradicating it completely through new mechanism or harvesting the innate immune response must continue [16–18]. Research to deal with every dwindling numbers of patients with less than ideal response or to reduce toxicity of therapy both in the short and long term is a “no brainer” as they say [19]. A reasonable balance between development costs return on investment, must also be achieved.

We do need to curb the cost of medications before the system implodes. This is not just a cml issue but is relevant to all diseases not just cancer, but adding drugs that are costlier but do not improve the results need to be examined. As treating physicians we should not be made to feel guilty that we are not giving our patients the “newest and the best”. Our patients who with the access to the internet, should not feel that they are getting inferior therapy if they do not get the new kid on the block or achieve the ideal response. When a representative of pharma talks to us or pays one of us to promote a product, they need to have damn good answers as to why we should be using it.

The other area that has seen major developments is disease monitoring. We have gone from the labor- intensive chromosome analysis to much more sensitive and mass applicable types of molecular testing that is in theory, available to all treating physicians. The issues here are first and foremost, making it available to the developing world for appropriate monitoring of treatment results. As I see it, we also need to put the most sensitive monitoring results in perspective, including alternate genetic findings and mutation analysis, so that they may truly become risk predictors, both for disease outcome and even for adverse events. By this I mean, not just finding them in patients who have done poorly, but also using them at diagnosis or treatment modification, to predict successful or unsuccessful options.

We have a lot for future thought and the battle is not over. This is just a summary list of some of the things that come to my mind.

1. Safer drugs
2. Ways of identifying, preventing and treating side effects—both the short term ones that studies have identified and appear in the product monographs and the long term ones.
3. Getting a lot more people to TFR eligibility
4. Successfully executing attempts at TFR
5. Identifying quickly those patients who need to go on to allografting
6. Making the newer drugs available to more people—both those who are payment challenged or in the developing world
7. Reducing the costs of the drugs so we do not break the bank. Sorry, pharma will always recoup their costs.
8. The elephant in the room—the economic adverse event
9. Placing drugs in their proper place—if no advantage why use the expensive cannons front line when peashooters might do.
10. Balancing chronic low-grade adverse events against potentially riskier events with other drugs.
11. Not all generics are the same especially once you get outside western world—standards for safety and efficacy must be established for all patients regardless of where they live.
12. More research on the CML stem biology with the hope of developing alternate targets
13. Revisiting the non-allograft immune modulation in CML
14. More research on global issues—why is the median age of diagnosis in the western world about 65 years, but go to the developing world, it is less than 40 years.

So, I now climb down from my soapbox, hopefully having left the reader with a few points to ponder. My career has spanned a phenomenal age of understanding the biology and therapy of a disease, something that only a few are lucky enough to have experienced. For that, I am grateful—for the changes, the interactions with colleagues who have made this a reality and for the patients who were the volunteers and the success stories. We need to remember that there are still hills to climb.
